# Genomic epidemiology of the Los Angeles COVID-19 outbreak and the early history of the B.1.43 strain in the USA

**DOI:** 10.1186/s12864-022-08488-7

**Published:** 2022-04-04

**Authors:** Longhua Guo, James Boocock, Evann E. Hilt, Sukantha Chandrasekaran, Yi Zhang, Chetan Munugala, Laila Sathe, Noah Alexander, Valerie A. Arboleda, Jonathan Flint, Eleazar Eskin, Chongyuan Luo, Shangxin Yang, Omai B. Garner, Yi Yin, Joshua S. Bloom, Leonid Kruglyak

**Affiliations:** 1 Department of Human Genetics, David Geffen School of Medicine, UCLA, Los Angeles, USA; 2 Howard Hughes Medical Institute, HHMI, Chevy Chase, USA; 3 Department of Biological Chemistry, David Geffen School of Medicine, UCLA, Los Angeles, USA; 4 Department of Pathology & Laboratory Medicine, David Geffen School of Medicine, UCLA, Los Angeles, USA; 5 Department of Psychiatry and Biobehavioral Sciences, David Geffen School of Medicine, UCLA, Los Angeles, USA; 6 Department of Computer Science, Samueli School of Engineering, UCLA, Los Angeles, USA; 7 Department of Computational Medicine, David Geffen School of Medicine, UCLA, Los Angeles, USA; 8 Octant, Inc, Los Angeles, USA

## Abstract

**Background:**

The severe acute respiratory syndrome coronavirus 2 (SARS-CoV-2) has caused global disruption of human health and activity. Being able to trace the early outbreak of SARS-CoV-2 within a locality can inform public health measures and provide insights to contain or prevent viral transmission. Investigation of the transmission history requires efficient sequencing methods and analytic strategies, which can be generally useful in the study of viral outbreaks.

**Methods:**

The County of Los Angeles (hereafter, LA County) sustained a large outbreak of severe acute respiratory syndrome coronavirus 2 (SARS-CoV-2). To learn about the transmission history, we carried out surveillance viral genome sequencing to determine 142 viral genomes from unique patients seeking care at the University of California, Los Angeles (UCLA) Health System. 86 of these genomes were from samples collected before April 19, 2020.

**Results:**

We found that the early outbreak in LA County, as in other international air travel hubs, was seeded by multiple introductions of strains from Asia and Europe. We identified a USA-specific strain, B.1.43, which was found predominantly in California and Washington State. While samples from LA County carried the ancestral B.1.43 genome, viral genomes from neighboring counties in California and from counties in Washington State carried additional mutations, suggesting a potential origin of B.1.43 in Southern California. We quantified the transmission rate of SARS-CoV-2 over time, and found evidence that the public health measures put in place in LA County to control the virus were effective at preventing transmission, but might have been undermined by the many introductions of SARS-CoV-2 into the region.

**Conclusion:**

Our work demonstrates that genome sequencing can be a powerful tool for investigating outbreaks and informing the public health response. Our results reinforce the critical need for the USA to have coordinated inter-state responses to the pandemic.

**Supplementary Information:**

The online version contains supplementary material available at 10.1186/s12864-022-08488-7.

## Introduction

Since the first report of pneumonia patients associated with a novel coronavirus in Wuhan, China in late December, 2019 [1], SARS-CoV-2 has spread across the globe, infecting 29 million people, and killing 927 thousand as of September 14th, 2020. The United States of America (USA) alone reported 194 thousand deaths [2]. Genomic surveillance via viral genome sequencing is crucial for determining outbreak dynamics, detecting viral evolution, and informing public health interventions. Studies in Washington State showed that most infections there likely stemmed from a single introduction event of strain WA1, followed by cryptic community transmission [3], while sequencing of viral genomes from Northern California and New York City demonstrated that there had been multiple independent introductions into these areas from international and domestic travelers [4, 5]. Viral genomes from samples collected during the period from March 22 to April 15 at the Cedars Sinai Medical Center also showed multiple introductions of SARS-CoV-2 into LA County [6].

The first complete genomes of SARS-CoV-2 were deposited into GISAID and GenBank in January, 2020 [1, 7]. Since then, large-scale global efforts to sequence SARS-CoV-2 led to 62,6441 genomes in GISAID as of July 14, 2020.

To facilitate the sequencing of SARS-CoV-2 genomes, we recently developed a rapid and inexpensive sequencing method based on targeted reverse transcription of the SARS-CoV-2 genome directly from patient RNA [8]. We used this method, together with a meta-transcriptomic approach, to generate 142 high-quality viral genome sequences from patients residing in LA County who were seen at UCLA Health. We used these genomes, together with publicly available ones, to investigate the early history of the SARS-CoV-2 outbreak in LA County.

## Results

### Rapid low-cost sequencing of SARS-CoV-2 genomes.

We recently developed V-seq, a sequencing method that uses virus-specific RT primers tiled across the SARS-CoV-2 genome for viral sequence enrichment [8]. The V-seq protocol is more rapid and 10 times cheaper than commercially available meta-transcriptomics approaches (*e.g.*, NEBNext Ultra II). We sequenced 122 patient samples from UCLA Health with V-seq and 138 samples with NEBNext Ultra II (Figure S1, Table S1). We obtained 97 and 63 high-quality genomes by V-seq and NEB, respectively (Figure S2). For both methods, samples with a higher amount of viral RNA, as determined by the cycling threshold (Ct) of the RT-qPCR used to detect the presence of the virus, had a higher fraction of reads aligning to SARS-CoV-2. To assess the accuracy of V-seq for variant identification, we compared high-confidence variant calls in 18 samples from which we recovered high-quality genomes with both methods. We did not find any discrepancies among 6,657 high-confidence genotype calls at 380 sites across the SARS-CoV-2 genome. These results showed that V-seq is a highly accurate approach for sequencing SARS-CoV-2 genomes.

### Multiple introductions of SARS-CoV-2 into LA County

**Fig. 1:**
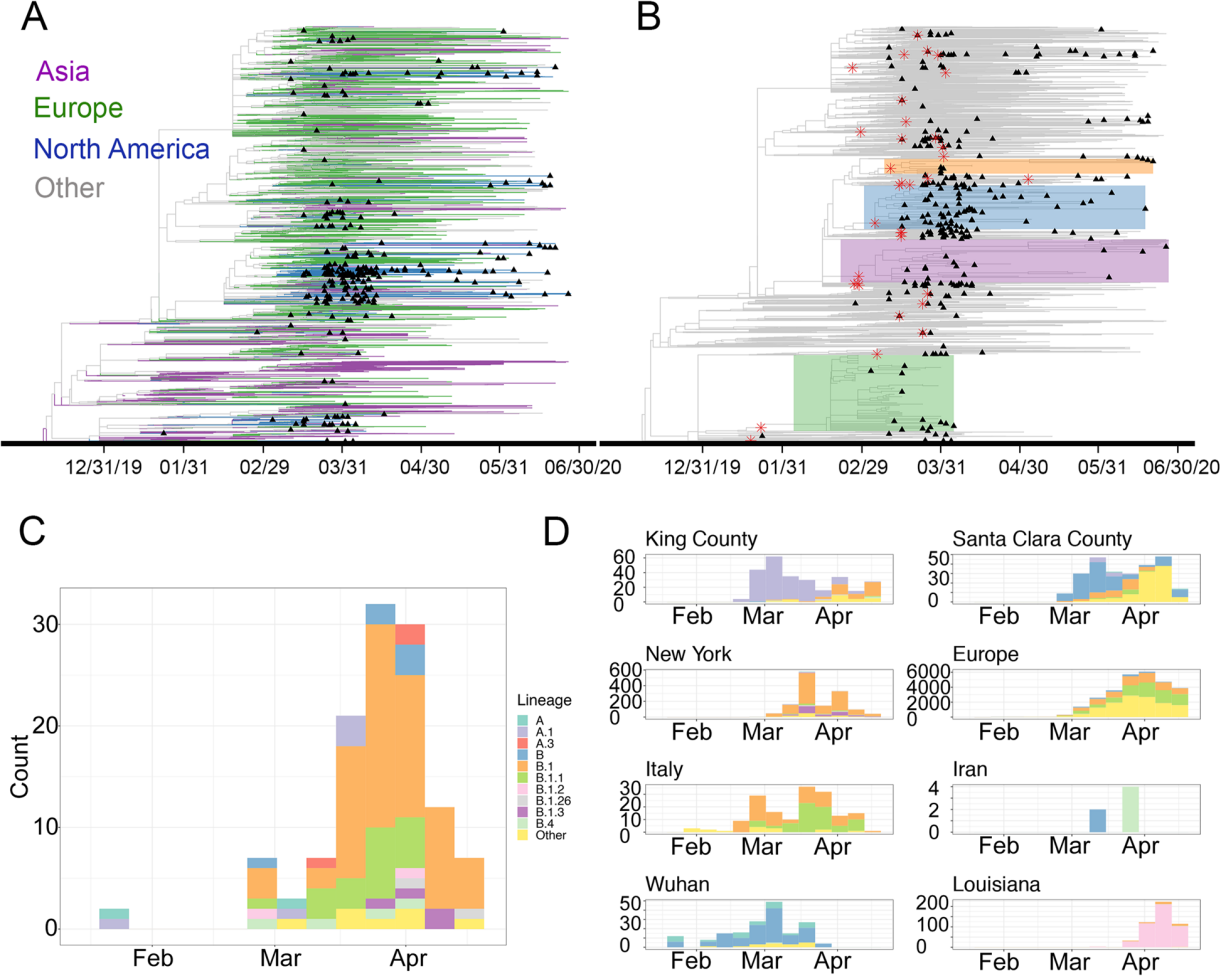
Multiple introductions of SARS-CoV-2 into LA county. **A** Phylogenetic tree of 286 LA county SARS-CoV-2 genomes together with 3,809 genomes sampled from across the world. Branches are colored according to the region of origin. Tip triangles (black) indicate the position of LA county sequences. The tree is rooted with strain Wuhan/Hu-1/2019. **B** The same tree as in A with different annotations. We zoomed in and highlighted four regions (blue, orange, purple, and green) of the tree where a single introduction was related to a cluster containing LA County genomes indicating community transmission. Nodes shown with a red star indicate a LA County introduction related to a cluster with more than one LA County genome. **C** Assignment of LA County introductions before April 19th, 2020 to lineages. **D** Observed frequency of different lineages from eight other COVID-19 hotspots from throughout the US and the world

We obtained 142 new SARS-CoV-2 genomes from samples collected in LA County between February 28 and June 22, 2020 (Figure S3). We combined these genomes with another 144 genomes from LA County obtained from GISAID on July 14, 2020. We performed a phylogenetic analysis with NextStrain using these 286 LA County genomes together with 3,809 genomes sampled from across the world [9]. LA County genomes were distributed throughout the resulting phylogenetic tree, consistent with multiple independent introductions (Figs. 1A, S4A). We used a parsimony-based approach to identify 145 distinct introductions of SARS-CoV-2 into LA county (Figs. 1B, S4B). One introduction was related to a large community outbreak cluster containing 58 LA County genomes, which we assigned to the US-specific lineage B.1.43. Thirty-three introductions were related to clusters with more than one LA County genome, and the remaining 111 introductions were found in clusters containing only a single LA County genome, with no evidence of community transmission in our sample.

We estimated that 122 introduction events occurred before April 19, 2020. We assigned these introductions to 17 distinct lineages related to global and early USA outbreaks (Fig. 1C-D, Table S2-3) [10]. Ninety-nine (81%) of these early introductions were assigned to European-derived lineages, whereas the remaining introductions were assigned to Chinese-derived lineages. The earliest introduction of SARS-CoV-2 in LA County involved the A lineage. Next, the derived A.1 lineage was introduced. This lineage was common in Washington State during the early outbreak in King County. At around the same time, the B lineage and its derivatives were introduced. We observed that different B lineages were introduced into LA County at around the time of the outbreak of each lineage in a geographic hotspot [10]. For example, B.1 was introduced into LA County during its outbreak in Italy and New York, while B.1.2 was introduced into LA County during its outbreak in Louisiana. These observations suggest that SARS-CoV-2 was repeatedly introduced into LA County by a diverse mix of domestic and international travelers.

### The history of a USA-specific SARS-CoV-2 lineage

**Fig. 2:**
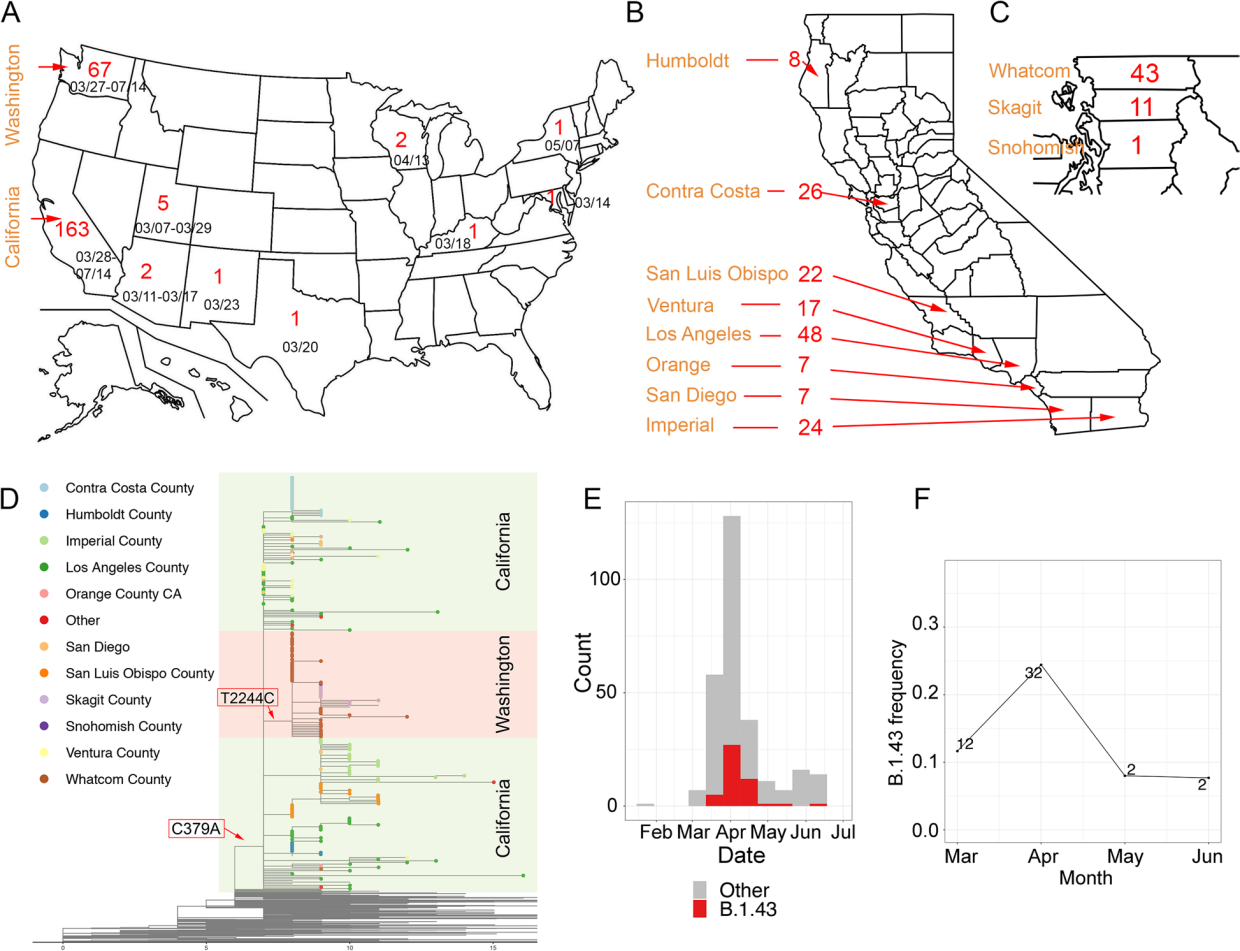
The history of the US-specific lineage B.1.43. **A** US map showing the states where the lineage B.1.43 has been found. Numbers are reported cases of B.1.43. CA: California; WA: Washington. **B-C** Map of California (B) and Washington State (C). Counties and the number of B.1.43 cases were labeled. **D** The B.1.43 strains found in Washington state (orange) are derived from the ancestral B.1.43 sequence by one mutation (T2244C). Some of the B.1.43 strains LA County (dark green) carried the ancestral haplotype. X-axis is the number of mutations compared to viruses in Wuhan, China. The nodes are colored based on the US counties from which they were collected. **E** The distribution of LA County genomes over time. **F** The frequency of B.1.43 in LA County summarized per month. The number of B.1.43 strains identified each month is written near the data point

Worldwide, a total of 247 B.1.43 samples, including our LA County genomes, were reported in GISAID as of July 14, 2020. Three were found outside the U.S. as early as March 17. Of the 244 USA B.1.43 samples, 67% were found in California and 28% were found in Washington State (Fig. 2A, Table S4). The remaining 5% were found in 8 states, including those that neighbor California or each other (Arizona, New Mexico, Utah and Texas) and those located elsewhere (New York, Maryland, Kentucky and Wisconsin). Of the 163 cases in California, 77% were found in LA and neighboring counties in Southern California, with the largest number of cases in LA County (N = 48) (Fig. 2B). Of the 67 samples from Washington State, 82% were found in three neighboring counties in Northern Washington, with the largest number of cases in Whatcom county (*N* = 43) (Fig. 2C). The other cases in Washington State did not have associated county information.

To gain insight into the origin of B.1.43 in the USA, we performed a phylogenetic analysis of the 247 B.1.43 and 987 other SARS-CoV-2 genomes sampled from around the world. B.1.43 lineages differ from B.1 at a single position, C379A (ORF1a, L64L) (Figs. 2D, S5). All 67 B.1.43 strains from Washington State had at least one additional derived mutation, with 65 sharing the same derived mutation, T2244C (ORF1a, V660A). Genomes with the ancestral B.1.43 sequence were identified only in LA County, three nearby counties (Ventura, San Diego, and Orange), Utah, Kentucky, and Australia. The earliest ancestral B.1.43 sequences were found in Utah on March 7, 2020 and in Southern California on March 28, 2020. The mutational signature and the geographical distribution of B.1.43 strains suggest that California or Utah might have been the source of the Washington State B.1.43 outbreak.

Longitudinal sampling of viral genomes in LA County from February to June allowed us to track the history of the B.1.43 lineage over time. We inferred that the B.1.43 lineage was introduced into LA County once, around March 7, 2020 (95% CI = March 4th, 2020—March 9th, 2020), and that following its introduction, its frequency among the circulating strains changed, peaking at ~ 25% in April and dropping to ~ 8% in May and June (Fig. 2E-F). This result suggests that the B.1.43 lineage was being replaced by other lineages introduced more recently.

### Genomic assessment of the effectiveness of local public health measures

**Fig. 3:**
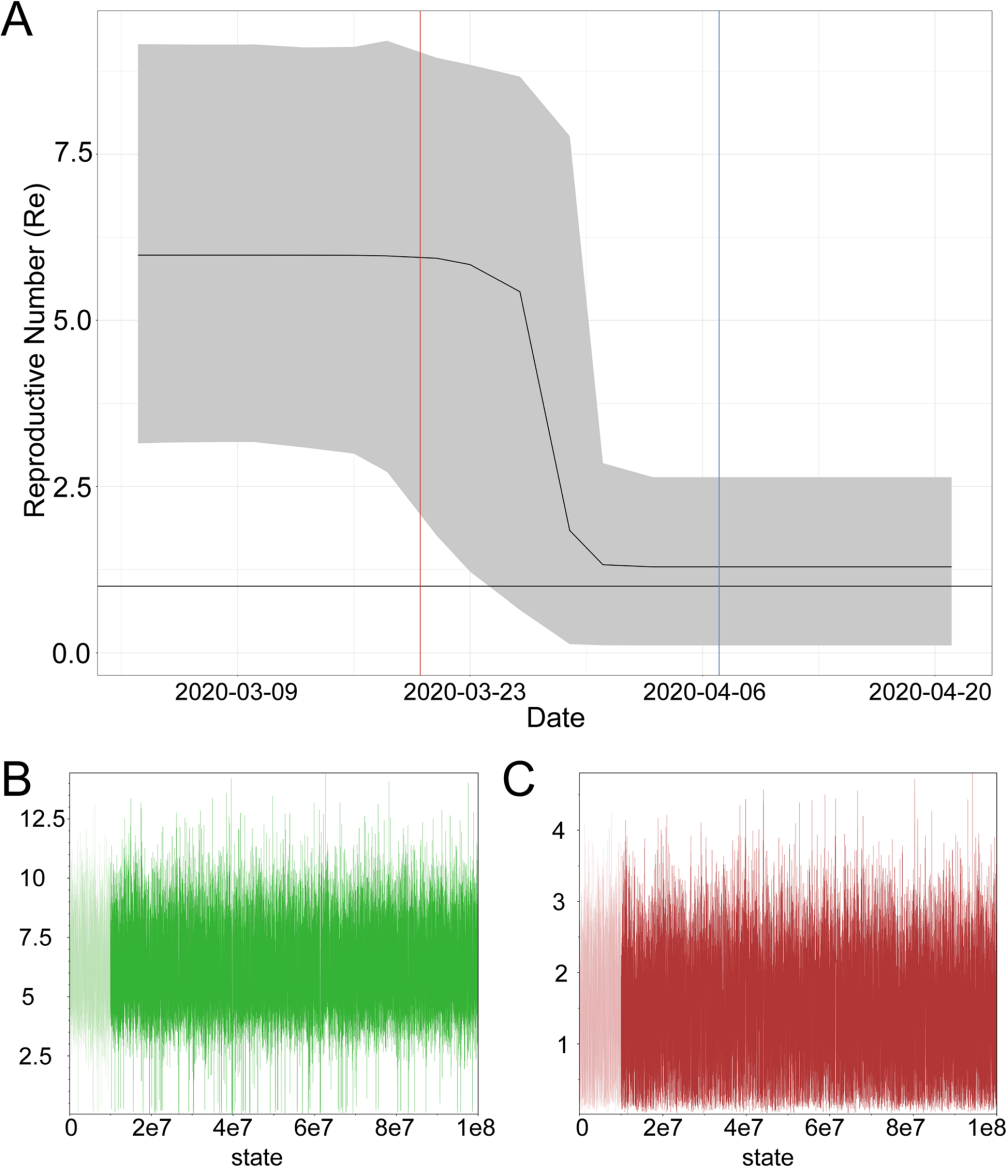
Genomic assessment of public health measures put in place to prevent the spread of SARS-CoV-2 in LA County. **A** Effective reproductive number (Re) quantified across time using all B.1.43 strains from LA County. These genomes were inferred to have arisen from a single introduction event. The grey ribbon shows the 95% highest posterior density (HPD) credible interval for our estimates of Re. The vertical lines depict the time when some public health measures were put in place in LA County. The ‘Safer at Home’ order issued for LA County put in place on March 20, 2020 (red). LA City required masks to be worn when visiting essential businesses on April 7th, 2020 (blue). **B-C** Convergence data for the two effective reproductive number parameters from one MCMC run. The y-axis shows the value of the effective reproductive number throughout the MCMC run. The x-axis is the state in the chain. **B** shows the parameter from the first time period. **C** shows the parameter for the second time period

To assess the effectiveness of public health measures put in place in LA County, we used a Bayesian analysis [11] of the LA County B.1.43 genomes to quantify changes in the rate of transmission of the B.1.43 lineage over time in LA County. The estimate of the effective reproductive number (Re) of this lineage rose to ~ 5.94 (95% CI = 3.1–9.3; Methods) in early March, but dropped to near 1 (95% CI = 0.14–2.8) by the middle of April (Fig. 3, Table S5). Mobility in Los Angeles County also decreased dramatically in April (https://covid19.apple.com/mobility). These results suggest that the “Safer at Home” order put into place in LA County on March 20, 2020, together with other social distancing measures, was effective at reducing the transmission of the virus.

## Discussion

We developed a faster and cheaper virus-targeted sequencing approach, V-seq, and applied it to SARS-CoV-2 samples from a major travel hub, LA County. Viral sequence variant identification by V-seq was as accurate as that by the commercial metagenomic kit. The V-seq protocol enabled more efficient high-coverage sequencing of large numbers of samples, which can accelerate genomic surveillance of viral outbreaks. We combined our 142 genomes with publicly available data and found that SARS-CoV-2 was introduced into LA County many times, likely via a variety of domestic and international travel routes. We studied the history of a USA-specific SARS-CoV-2 lineage, B.1.43, by combining mutational signatures and regional distributions, and found evidence that B.1.43 originated in Southern California or Utah and spread to northern Washington State.

A limitation of our study is that we partially relied on publicly available genome sequences for our inferences. Publicly available genomes were sampled at different rates throughout the USA and the world. As an example, the lack of B.1.43 lineages outside Washington State and California could reflect a lack of sequencing data from other states. In agreement with another study of LA County genomes [6], we found evidence that SARS-CoV-2 was introduced many times. However, without detailed travel information, we could not pinpoint the sources of these introductions or rule out community transmission post-introduction. Finally, the UCLA patient population is affluent relative to all of LA County, and likely to travel more frequently, potentially resulting in differences between lineage frequencies observed in our sample and those in LA County, as well as in overestimation of the role of multiple introductions in the overall dynamics of the SARS-CoV-2 outbreak in LA County.

Early in the pandemic, LA County officials followed the advice of public health experts. Schools, bars, and gyms were closed on March 16, 2020, and all non-essential business activity was stopped on March 20, 2020. After these orders were put in place, the number of reported daily cases continued to increase, with an average of ~ 850 cases per day in April and May (LA Times’ independent count; https://github.com/datadesk/california-coronavirus-data). However, the relationship between the timing of the orders and the increase in case numbers could be complicated by reporting delays, changes in testing practices, and incubation times. We analyzed the rate of transmission of SARS-CoV-2 in LA County using the genome sequences, and found evidence that the public health measures were effective in reducing the transmission of the virus. SARS-CoV-2 was repeatedly introduced into LA County from hotspot regions throughout the USA and the world [10]. These ongoing introductions may have undermined the effectiveness of the control measures put in place in LA County [12]. Our assessment of the effectiveness of the public health measures is based on one single SARS-CoV-2 lineage, B.1.43, which was the only strain in the early LA County outbreak that had sufficient longitudinal coverage in our dataset. This limitation may bias our assessment. Nonetheless, the assessment is supported by publicly available mobility data. Our results reinforce the critical need for the USA to coordinate local responses to the SARS-CoV-2 pandemic.

## Materials and methods

### Sample collection and processing

The clinical samples were submitted to be tested for SARS-CoV-2 at the Virology laboratory at UCLA between Feb 21, 2020 and June 28th, 2020. The samples were tested on one of three diagnostic testing protocols approved for Emergency Use Authorization (EUA) by the Food and Drug Administration (FDA). The three protocols were: CDC 2019-Novel Coronavirus (2019-nCoV) Real-Time RT-PCR Diagnostic Panel, DiaSorin Molecular Simplexa™ COVID-19 Direct or TaqPath COVID-19 Combo Kit. The extracted RNA from these samples were approved to be sequenced by UCLA’s Institutional Review Board (IRB) under studies IRB#20–000,527 and IRB#20–001,157. Extracted mRNA from patient samples were used for library construction with V-seq or NEBNext® Ultra™ II RNA Library Prep Kit (New England Laboratory, E7770L).

### Raw read processing and alignment

All libraries were sequenced on an Illumina NextSeq 500 Sequencing System. We used bcl2fastq (v2.20.0.422) to obtain libraries for each sample allowing one barcode mismatch for NEB Ultra II samples, and 0 barcode mismatch for V-seq libraries.

For the V-seq libraries, we removed all custom RT-primers using a custom script written in the R (v4.0.0) programming language [13]. This script uses the ShortRead (v1.46) [14] package from Bioconductor [15]. We mapped the reads from each library to a composite reference genome consisting of human (hg38) and SARS-CoV-2 (NC_045512) using the bwa (v0.7.17-r1188) mem command [16]. For the V-seq libraries from primer sets oP1, oP3 or oP4, we combined the 3 base-pair unique molecular identifier (UMI) with the 6 base random or “not-so-random” hexamer sequence to create a 9 base-pair UMI. For reads assigned to primer set oP2, we combined the 8 base-pair UMI with the 6 base-pair random hexamer to create a 14 base-pair UMI. For a more detailed description of the primer design see [8]. We used the GroupReadsByUmi tool from the fgbio (v1.2.0; https://github.com/fulcrum-genomics/fgbio) toolkit to group reads using this UMI. We generated molecular consensus sequences using the fgbio CallMolecularConsensusReads tool. For the NEB libraries, PCR duplicates were removed using MarkDuplicates from the Picard tool suite (v2.22.2; (http://broadinstitute.github.io/picard). We calculated the number of reads that mapped to human rRNA, other regions of the human genome, and SAR2-CoV-2 before and after deduplication. We visualized the relationship of these metrics to the cycling threshold (Ct) of the RT-qPCR used to detect the presence of SARS-CoV-2 in each patient sample using ggplot2 (v3.3) [17].

### Variant calling and consensus sequence generation

We merged reads across unique patient sample and library type combinations and called bases at all sites in each of these samples using the mpileup and call commands of bcftools (1.10.2) [18]. We removed any sites with depth less than 3 or a variant quality (QUAL) of less than 20. We flagged any site called heterozygous in at-least one sample, and calculated the allelic ratio of the alternative allele to the total depth in each sample for these variants. If the allelic ratio was between > 0.1 and < 0.9 and had at least two unique reads supporting each allele, we flagged the variant as being a possible intra-patient variable site. We removed any samples with greater than 4 called intra-patient variable sites. We used bcftools to create consensus sequences and masked any bases that were not found in the filtered VCF file. We also masked any heterozygous sites. Consensus sequences with greater than 80% coverage at a depth of > 3 were considered to have passed quality control.

### Phylogenetic analysis

The available SARS2-CoV-2 genomes were downloaded from GISAID (Accessed July 13th, 2020) [19, 20]. We filtered these genomes using the Nextstrain pipeline [9]. We required these genomes to be at least 25,000 bases in length and have at most 4,500 bases of missing data. We also removed a sequence (USA/CA-ALSR-0513-SAN/2020) which had an incorrect date recorded in GISAID. These filtering steps left us with 59,830 genomes. We assigned lineages to the UCLA Health and publicly available genomes according to a recently proposed nomenclature with Pangolin (https://github.com/cov-lineages/pangolin) [21].

We performed phylogenetic analysis of all LA County genomes using Nextstrain (v1.16.7) [9]. In more detail, we combined all LA County genomes with a sampling of genomes from around the world. To achieve this, we utilized proximity sampling and allowed 20 samples per country, year, and month combination, and 10 contextual samples per country and year combination (see https://nextstrain.github.io/ncov/ for a more detailed description of how sampling works in Nextstrain). These genomes were run through the entire Nextstrain pipeline, and we explored the results and exported the trees from the Auspice web application (v2.16.0). For our focused analysis of the B.1.43 lineage, we combined all genomes assigned to this lineage with a random sampling of genomes from around the world. As before, we utilized proximity sampling but only allowed 2 samples per country and year combination. We also sampled contextually 2 samples per country and year combination.

To identify the SARS-CoV-2 introduction events in Los Angeles County, we utilized maximum parsimony as implemented in the Castor (v1.6.2) package to infer the value of a two-state character (LA County vs. non-LA County) for every node in the tree [22]. When the child of a node was assigned to LA County, but the parent was non-LA County, we determined that an introduction event must have happened. We set all ambiguous assignments to non-LA County. We set all polytomies (nodes with greater than two genomes) to non-LA County and any children of this node assigned to LA County were determined to be independent introductions. We assigned lineages to introductions by taking the most common lineage found in the offspring of these nodes.

### Phylodynamics analysis

To investigate how the transmission of SARS-CoV-2 changed overtime in LA County, we used a Bayesian birth–death skyline model implemented in BEAST (v2.5) [11]. For this analysis, we used the genomes from the cluster of B.1.43 strains found in LA County that we inferred arose from a single introduction event collected between the 24th of February to the 19th of April, 2020. The HKY + $\Gamma$ model of nucleotide substitutions was used with a strict molecular clock. This dataset did not display a strong temporal signal prompting us to use an informative prior reflecting recent estimate for the mutation rate of SARS-CoV-2 [23]. This clock rate had a $\Gamma$ prior distribution with a mean of $8 \cdot 1{0}^{-4}$ subs/site/year and a standard deviation of $5 \cdot 1{0}^{-4}$ reflecting estimates for the mutation rate of SARS-CoV-2. We assumed that the infectious period was 10 days, which is in line with epidemiological estimates [24]. We set the model up to return effective reproductive number (Re) for 2 time intervals, as determined from the model. We used Markov Chain Monte Carlo (MCMC) to estimate the parameters of the model with a chain-length of 10e8 and sampling every 5000 steps. We removed the first 10% of the chain as burnin. We assessed the sampling of the trees using Tracer (v 1.7.1) and made sure that our 2 effective reproductive number (Re) parameters had an effective sample size of at least 200. This approach is similar to a study of New Zealand SARS-CoV-2 genomes [25]. For this analysis, we removed a sequence (USA/CA-CSMC31/2020), which caused the initial tree to be unrealistically long and prevented the model from obtaining realistic estimates for when the outbreak started, leaving us with 45 B.1.43 genomes for this analysis. We performed three independent runs of MCMC and confirmed that the parameters converged each time.

### Tests of convergence

We performed the Geweke’s test and Gelman-Rubin test [26–28] on our MCMC chains to assess convergence. To perform these tests, we utilized the R package coda (v0.19). We applied Geweke’s test to our original chain and three replicate runs. In all cases, the Z-statistic suggested that the estimated parameters were converged (P > 0.05). We applied the Gelman-Rubin test to compare all three replicate runs. We obtained scale reduction factors of between 1.0 and 1.01 for every parameter, which provides evidence that the chains had converged. These convergence statistics support our visual inspections of the MCMC chains where we made the conclusion that the chains had converged.

## Supplementary Information

**Additional file 1: Table S1.** Collection dates and quality control for 260 patient samples.

**Additional file 2: Table S2.** Lineage assignment for 142 high quality patient samples from UCLA health.

**Additional file 3: Table S3.** Lineage assignment for global SARS-CoV-2 samples.

**Additional file 4: Table S4.** B.1.43 lineages found among the GISAID and UCLA Health SARS-CoV-2 genomes.

**Additional file 5: Table S5.** Parameters for effective reproductive number estimation.

**Additional file 6: Figure S1.** Contrasting QC metrics between V-seq and meta-transcriptomics. A) Coverage of the SARS2-CoV-2 genome at >=3x compared to the cycling threshold (Ct). The horizontal line in black represents the cut-off for determining whether genomes passed quality-control. B) Proportion of reads mapping confidently to SARS-CoV-2 before removing PCR duplicates compared to the Ct. C) Read duplication rate for reads mapped to SARS-CoV-2 for both NEB and V-seq libraries. D) Proportion of reads mapping confidently to SARS-CoV-2 after removing PCR duplicates. **Figure S2.** Summary of samples collected for sequencing using both V-seq and NEB approaches. A) Histogram of collection dates colored by whether they were sequenced using NEB or V-seq methods. B) Histogram of collection dates colored by whether the genome passed QC. C) Ct values for genomes passing and not passing QC sequencing using NEB and V-seq. **Figure S3.** Collection dates of high-quality Los Angeles County SARS-CoV-2 genomes from UCLA Health. **Figure S4.** Multiple introductions of SARS-CoV-2 into LA county. A) Phylogenetic tree of 286 LA County SARS-CoV-2 together with 3,809 genomes sampled from across the world. Branches are colored according to the region of origin. Tip triangles (black) indicate the position of LA county sequences on the tree. In B) is the same tree with different annotations. We zoomed in and highlighted four regions (blue, orange, purple, and green) of the tree where a single introduction was related to a cluster containing LA County genomes indicating community transmission. Nodes shown with a red star indicate a LA County introduction related to a cluster with more than one LA County genome. The x-axis is in units of mutations away from the root (NC_045512). **Figure S5.** Phylogenetic tree of 247 B.1.43 and 987 other SARS-CoV-2 genomes from around the world built using Nextstrain. We have zoomed in on the clade containing all B.1.43 sequences and highlighted the tips according to the county of origin. 6 branches are labelled according to their ancestral mutation. The x-axis shows the sampling dates.

**Additional file 7. **

## Data Availability

Analysis code and processing scripts, and snakemake (v5.17.0) pipelines for our analysis are available at https://github.com/theboocock/COVID-NGS2. The data that is necessary for generating the figures and tables can be found at Data Dryad (https://doi.org/10.5068/D1H102). Virus genome sequences were uploaded to GISAID (https://gisaid.org). Accession ID and genome sequences are available in supplemental files.
