# Supplementary material for: Genomic epidemiology of the Los Angeles COVID-19 outbreak and the early history of the B.1.43 strain in the USA
Source: BMC Genomics. 2022 Apr 4;23:260. doi: 10.1186/s12864-022-08488-7 (PMC8978495; doi:10.1186/s12864-022-08488-7)
Supplement: Supplementary file 6 — Additional file 6: Figure S1. Contrasting QC metrics between V-seq and meta-transcriptomics. A) Coverage of the SARS2-CoV-2 genome at >=3x compared to the cycling threshold (Ct). The horizontal line in black represents the cut-off for determining whether genomes passed quality-control. B) Proportion of reads mapping confidently to SARS-CoV-2 before removing PCR duplicates compared to the Ct. C) Read duplication rate for reads mapped to SARS-CoV-2 for both NEB and V-seq libraries. D) Proportion of reads mapping confidently to SARS-CoV-2 after removing PCR duplicates. Figure S2. Summary of samples collected for sequencing using both V-seq and NEB approaches. A) Histogram of collection dates colored by whether they were sequenced using NEB or V-seq methods. B) Histogram of collection dates colored by whether the genome passed QC. C) Ct values for genomes passing and not passing QC sequencing using NEB and V-seq. Figure S3. Collection dates of high-quality Los Angeles County SARS-CoV-2 genomes from UCLA Health. Figure S4. Multiple introductions of SARS-CoV-2 into LA county. A) Phylogenetic tree of 286 LA County SARS-CoV-2 together with 3,809 genomes sampled from across the world. Branches are colored according to the region of origin. Tip triangles (black) indicate the position of LA county sequences on the tree. In B) is the same tree with different annotations. We zoomed in and highlighted four regions (blue, orange, purple, and green) of the tree where a single introduction was related to a cluster containing LA County genomes indicating community transmission. Nodes shown with a red star indicate a LA County introduction related to a cluster with more than one LA County genome. The x-axis is in units of mutations away from the root (NC_045512). Figure S5. Phylogenetic tree of 247 B.1.43 and 987 other SARS-CoV-2 genomes from around the world built using Nextstrain. We have zoomed in on the clade containing all B.1.43 sequences and highlighted the tips according to th [file 12864_2022_8488_MOESM6_ESM.pdf]

Supplementary Figures

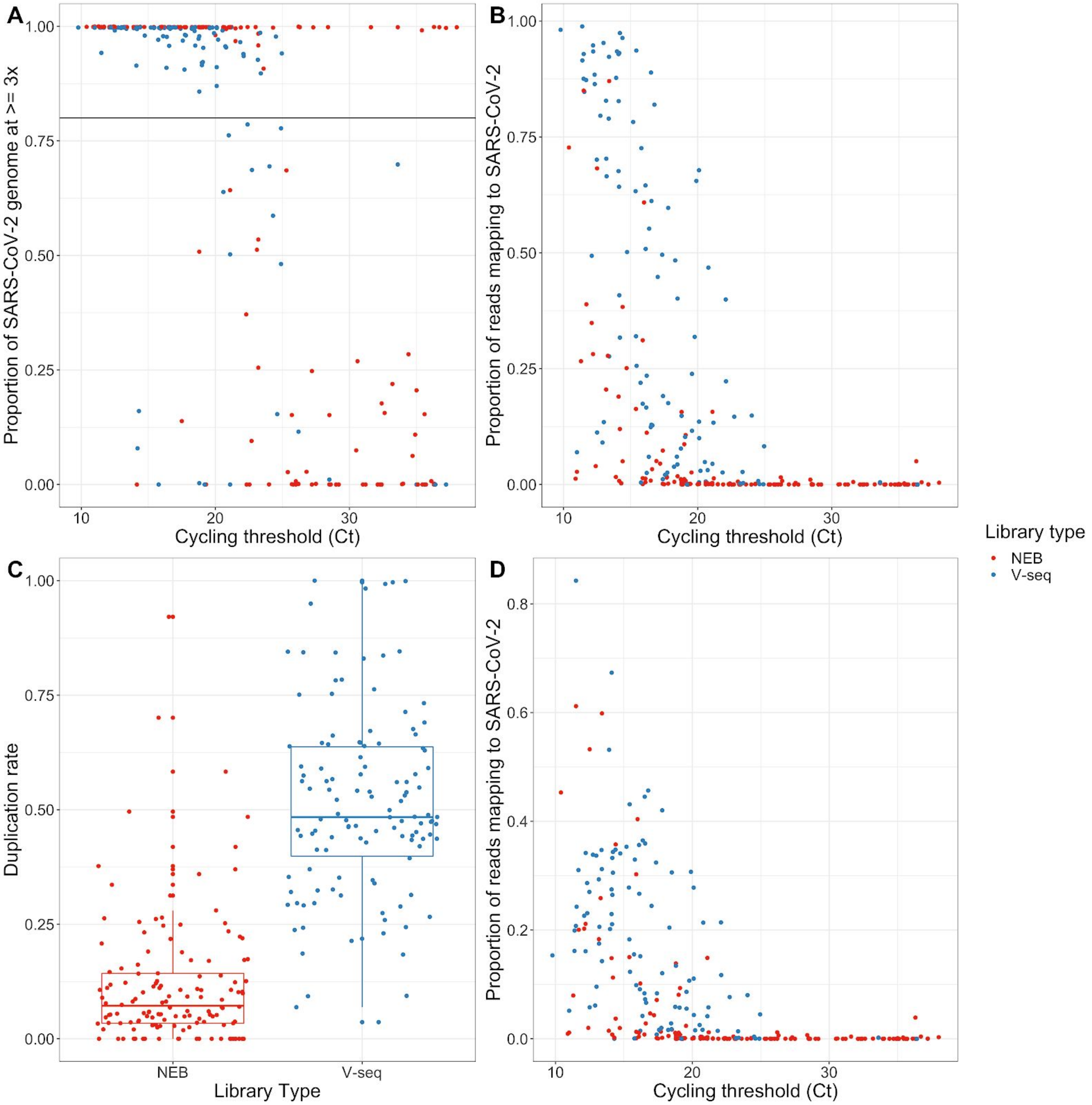

Figure S1: Contrasting QC metrics between V-seq and meta-transcriptomics. **A**) Coverage of the SARS2-CoV-2 genome at >=3x compared to the cycling threshold (Ct). The horizontal line in black represents the cut-off for determining whether genomes passed quality-control. **B)** Proportion of reads mapping confidently to SARS-CoV-2 before removing PCR duplicates compared to the Ct. **C)** Read duplication rate for reads mapped to SARS-CoV-2 for both NEB and V-seq libraries. **D)** Proportion of reads mapping confidently to SARS-CoV-2 after removing PCR duplicates.

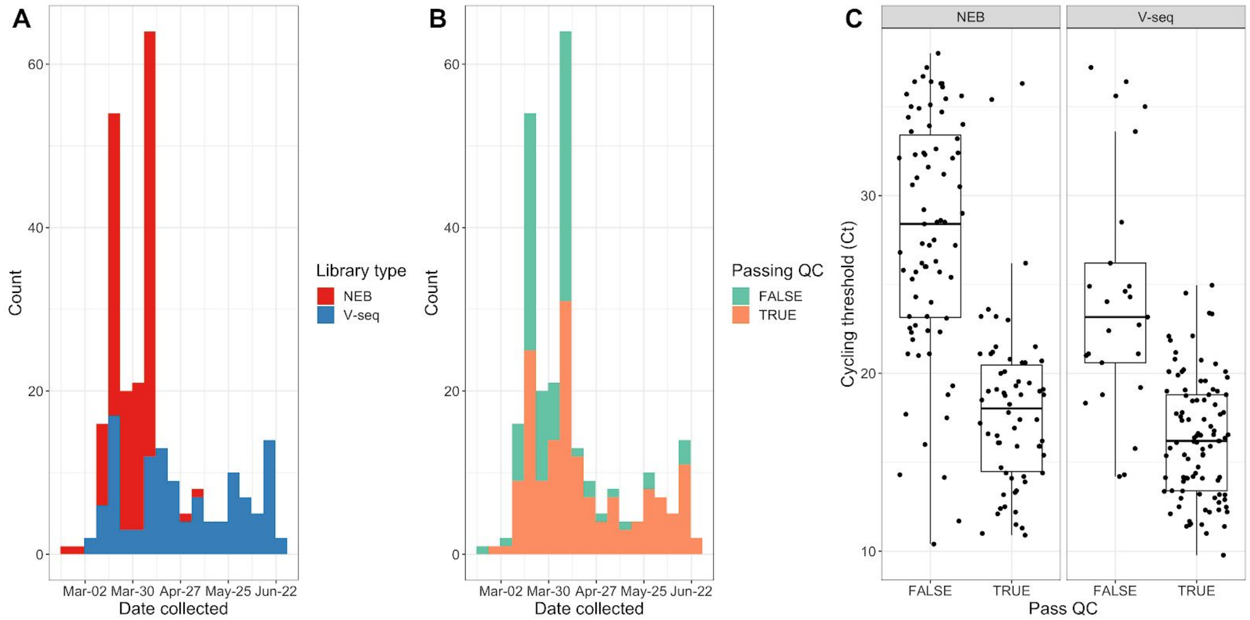

Figure S2: Summary of samples collected for sequencing using both V-seq and NEB approaches. **A)** Histogram of collection dates colored by whether they were sequenced using NEB or V-seq methods. **B)** Histogram of collection dates colored by whether the genome passed QC. **C)** Ct values for genomes passing and not passing QC sequencing using NEB and V-seq.

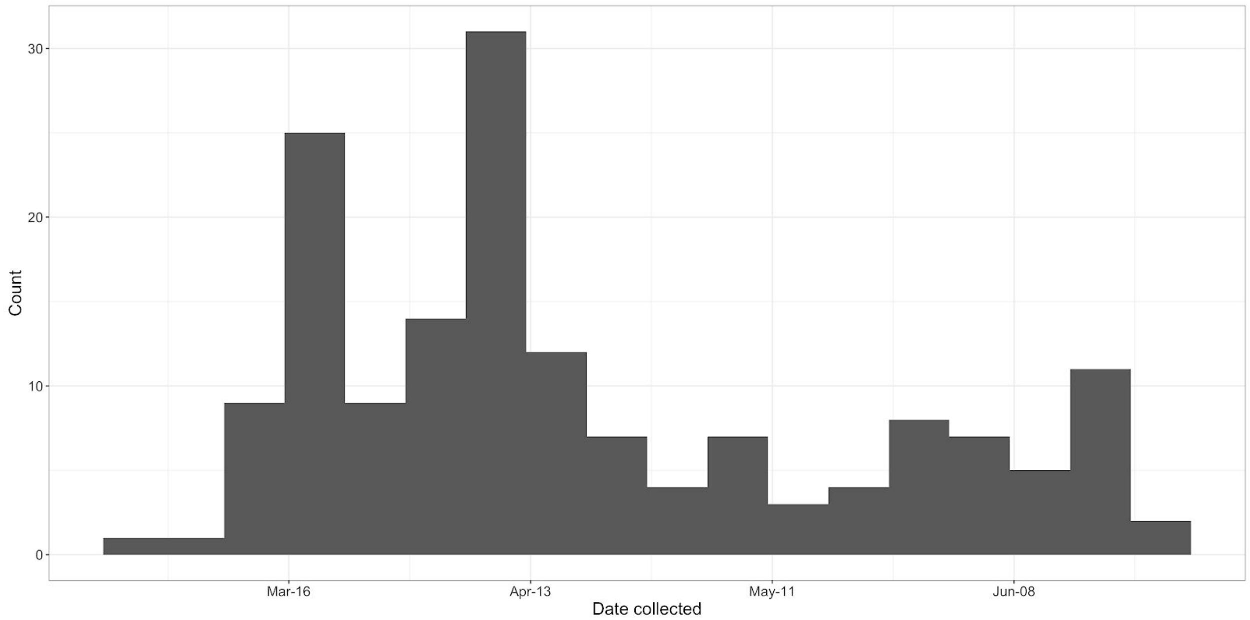

Figure S3: Collection dates of high-quality Los Angeles County SARS-CoV-2 genomes from UCLA Health.

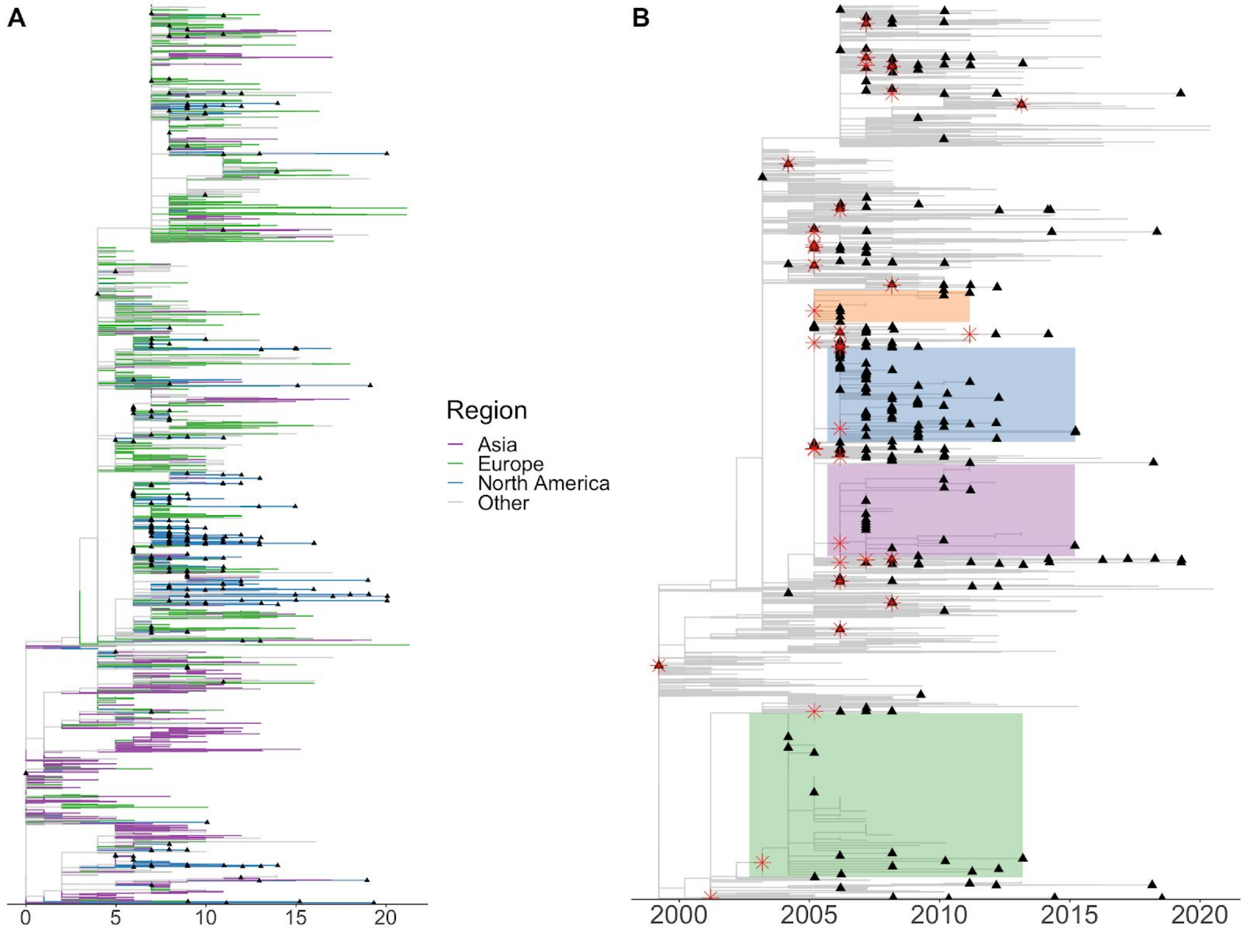

Figure S4: Multiple introductions of SARS-CoV-2 into LA county. A) Phylogenetic tree of 286 LA County SARS-CoV-2 together with 3,809 genomes sampled from across the world. Branches are colored according to the region of origin. Tip triangles (black) indicate the position of LA county sequences on the tree. In **B)** is the same tree with different annotations. We zoomed in and highlighted four regions (blue, orange, purple, and green) of the tree where a single introduction was related to a cluster containing LA County genomes indicating community transmission. Nodes shown with a red star indicate a LA County introduction related to a cluster with more than one LA County genome. The x-axis is in units of mutations away from the root (NC_045512).

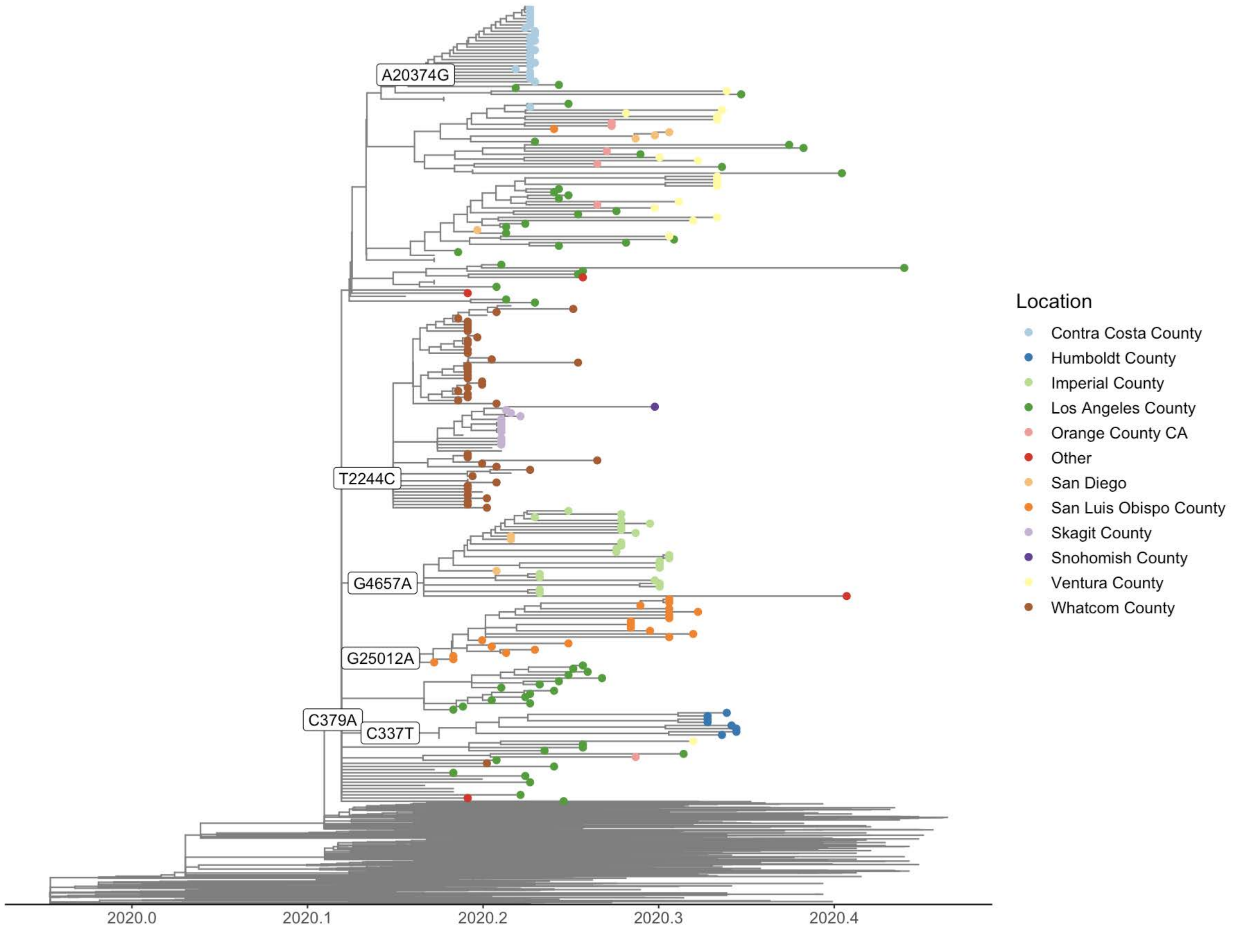

Figure S5: Phylogenetic tree of 247 B.1.43 and 987 other SARS-CoV-2 genomes from around the world built using Nextstrain. We have zoomed in on the clade containing all B.1.43 sequences and highlighted the tips according to the county of origin. 6 branches are labelled according to their ancestral mutation. The x-axis shows the sampling dates.
